# Catheter-Related ESBL-Producing *Leclercia adecarboxylata* Septicemia in Hemodialysis Patient: An Emerging Pathogen?

**DOI:** 10.1155/2020/7403152

**Published:** 2020-01-21

**Authors:** Roaa Sultan Alosaimi, Mai Muhmmed kaaki

**Affiliations:** ^1^Department of Medicine, Ministry of Health, Jeddah, Saudi Arabia; ^2^Department of Microbiology, King Abdul-Aziz Medical City, Jeddah, Saudi Arabia

## Abstract

We report a multidrug-resistant strain of *Leclercia adecarboxylata* which was responsible for a catheter-related bacteremia, in a 50-year-old female with an end-stage renal disease on hemodialysis. The isolated strain was an extended beta-lactamase producer. Based on a literature review of *L. adecarboxylata*, there have been only two reports of extended beta-lactamase producer strains. To our knowledge, this is the first case reported in Saudi Arabia.

## 1. Introduction

*Leclercia adecarboxylata* is a motile, Gram-negative, oxidase-negative bacterium, which was first described by Leclerc in 1962 as *Escherichia adecarboxylata* [1]. Based on nucleic acid and protein electrophoretic techniques, “*E. adecarboxylata*” was separated from the “*Enterobacter agglomerans*” complex [2] to which it was assigned temporarily and renamed *Leclercia adecarboxylata* [3]. This bacterium was isolated from environmental samples, including water and soil [4]. However, a cumulative report of human infections by *L. adecarboxylata* mostly in immunocompromised patients suggested that the organism is an opportunistic bacterium and the infections caused by it are underestimated. It has been reported to cause septicemia [5], wound infection [6, 7], urinary tract infection [8, 9], posttraumatic polymicrobial infection [10], soft tissue infection [11], and peritonitis [12]. The majority of clinical isolates of *L. adecarboxylata* were susceptible to commonly used antibiotics [4], and multidrug-resistant isolates have rarely been reported [8]. Here, we report a case of extended-spectrum beta-lactamase-producing *L. adecarboxylata* from a patient with catheter-related septicemia.

## 2. Case Report

A 50-year-old female patient with a known case of diabetes mellitus and end-stage renal disease due to hypertension was started on hemodialysis in March 2014. Permanent tunneled catheters were placed into the right internal jugular vein as the patient was not fit for AVF creation due to vascular occlusions.

On 7 April 2019, at the end of a dialysis session, the patient developed fever (39°C) and chills. Fever was documented at 39°C, was associated with chills and rigors, and was not responding to antipyretics. In the emergency department, the patient reported no change in weight, loss of appetite, or night sweats. She denied any history of respiratory, gastrointestinal, neurological symptoms. On examination, the temperature was 39°C, the blood pressure 150/75 mm Hg, the heart rate 110 beats per minute, and the respiratory rate 18 breaths per minute. The patient was fully conscious and oriented. The remainder of the general medical examination was normal.

She was admitted to our nephrology unit with possible source catheter-related blood stream infection. She was started on ceftazidime 2 gm IV once daily and vancomycin 1 gm IV Q HD as per the protocol. The white blood cell count was 13.3 × 10^3^ cells, C-reactive protein was 278 mg/dl, and procalcitonin was 52.27. The electrocardiogram revealed sinus tachycardia, and chest radiography was negative.

Blood culture from venous and central catheters was sampled separately, and the causative microorganism was identified as a Gram-negative, motile, lactose-fermenting bacillus, which was identified by the Vitek MS automatic identification system using a GN card as *Leclercia adecarboxylata*. Antibiotic sensitivity testing using Vitek 2 showed that it is an extended-spectrum beta-lactamase producer. The results are summarized in Table 1 (https://www.ncbi.nlm.nih.gov/pmc/articles/PMC3421816/table/T1/). The *L. adecarboxylata* strain was resistant to most beta-lactams, including narrow, expanded, and broad-spectrum cephalosporins. But it was susceptible to all quinolones and carbapenems tested.

The treatment regimen was changed to meropenem 500 mg IV once daily and gentamicin based on antibiotic susceptibility tests (ASTs) from blood cultures. This targeted therapy was successful, and the patient became afebrile. The patient received meropenem and gentamicin for two weeks, and then she was discharged in good condition.

## 3. Discussion

*L. adecarboxylata* is a Gram-negative bacillus belonging to the Enterobacteriaceae family. The association of *L. adecarboxylata* with catheter (particularly tunneled CVC) related septicemia is increasingly reported [13]. In 2013, De Mauri and colleagues reported that there were 15 reports of adult patients with catheter-related septicemia due to *L. adecarboxylata* [5]. Bacterial resistance to antibiotics is increasing worldwide in healthcare settings and in the community. The dissemination of extended-spectrum beta-lactamase Enterobacteriaceae (ESBL-E) is alarming [14]. Antibiotic-resistant *L. adecarboxylata* strains have been reported in six different cases. Of these cases, only two were extended beta-lactamase producer isolates. The first case from a patient with acute myeloid leukaemia produced ESBL. This strain encoded SHV-type beta-lactamases [15]. The second case was ESBL producing a multidrug-resistant *L. adecarboxylata* strain harbouring blaTEM-1, blaCTX-M-3, and intl1 cassette (dfrA12-orfF-aadA2) genes in a 47-year-old female with breast cancer [16]. Although *L. adecarboxylata* has been recognized as a relatively insignificant human pathogen due to its low virulence and high antibiotic susceptibility, multidrug-resistant strains can become life-threatening human bacterial pathogens by acquiring genetic determinants, including blaSHV, blaTEM-1, blaCTX-M group 1, and intl1 genes. Of note, *L. adecarboxylata* is an uncommon isolate in microbiology laboratories, but it will likely be more often identified with new diagnostic techniques.

In conclusion, we report the third case of catheter-related septicemia due to ESBL producing a multidrug-resistant *L. adecarboxylata* strain in a 50-year-old female with end-stage renal disease.

## Figures and Tables

**Table 1:** *Leclercia adecarboxylata* antibiotic susceptibility profile.

Antimicrobial agents	MIC (ug/ml)^*∗*^	Susceptibility
Amoxicillin/clavulanate	4	R
Ampicillin	≥32	R
Ceftriaxone	≥64	R
Ciprofloxacin	≤0.25	S
Gentamicin	≤1	S
Amikacin	≤2	S
Meropenem	≤0.25	S
Trimethoprim/sulfamethoxazole	≤20	S
Piperacillin/tazobactam	≤4	R

MIC = minimum inhibitory concentration; S = sensitive; R = resistant. ^*∗*^Vitek 2 automatic system using an AST GN card.

## Data Availability

The authors declare that this article does not show the name or data of the patient.
